# People Living with HIV Easily lose their Immune Response to SARS-CoV-2: Result From A Cohort of COVID-19 Cases in Wuhan, China

**DOI:** 10.21203/rs.3.rs-543375/v1

**Published:** 2021-06-07

**Authors:** Yanbin Liu, Yanling Xiao, Songjie Wu, Gifty Marly, Fangzhao Ming, Xiaoya Wang, Mengmeng Wu, Ling Feng, Weiming Tang, Ke Liang

**Affiliations:** Zhongnan Hospital of Wuhan University; Zhongnan Hospital of Wuhan University; Zhongnan Hospital of Wuhan University; Nanjing Medical University; Wuchang District Center for Disease Control and Prevention; People’s Hospital; Zhongnan Hospital of Wuhan University; Zhongnan Hospital of Wuhan University; Dermatology Hospital of Southern Medical University; Zhongnan Hospital of Wuhan University

**Keywords:** SARS-CoV-2, COVID-19, People living with HIV (PLWH), Immune response

## Abstract

**Background:**

To date, whether the immune response for SARS-CoV-2 infection among people living with HIV(PLWH) is different from HIV-naïve individuals is still not clear.

**Methods:**

In this cohort study, COVID-19 patients admitted to hospital in Wuhan between January 15 and April 1, 2020, were enrolled. Patients were categorized into PLWH and HIV-naïve group. All patients were followed up regularly (every fifteen days) until November 30, 2020, and the immune response towards SARS-CoV-2 was observed.

**Results:**

Totally, 18 PLWH and 185 HIV-naïve individuals with COVID-19 were enrolled. The positive conversion rates of IgG were 56% in PLWH and 88% in HIV-naïve patients respectively, and the peak was on the 45th day after COVID-19 onset. However, the positive rate of IgG dropped to 12% in PLWH and 33% among HIV-naïve individuals by the end of the study. The positive conversion rate of IgG among asymptomatic carriers is significantly lower than that among moderate patients (AOR = 0.18, 95% CI: 0.05–0.65) and PLWH had a lower IgG seroconversion rate compared to the HIV-naive group (AOR = 0.22, 95% CI: 0.05–0.90). Patients with lower lymphocyte counts at onset had a higher positive conversion rate (AOR = 0.29, 95% CI: 0.09–0.90) and longer duration for IgG (AHR = 4.01, 95% CI: 1.78–9.02).

**Conclusions:**

The positive conversion rate of IgG for SARS-CoV-2 was relatively lower and quickly lost in PLWH, which meant PLWH was in a disadvantaged situation when affected with COVID-19.

## Background

The 2019 coronavirus disease (COVID-19) which is knowingly caused by the severe acute respiratory syndrome coronavirus 2 (SARS-CoV-2) has a strong global impact in the year 2020, and its impact is still ongoing[1]. However, to date, our comprehensive understanding of immune response for SARS-CoV-2 infection is still questionable as clinical findings continue to contradict each other [2–4].

For example, a study in Iceland concluded that antibodies for SARS-CoV-2 did not decline within four months after diagnosis [2]. In direct contrast, other comparative studies invariably observed a substantial decrease in antibodies overtime after infection [3, 4], the last study in Wuhan also revealed the antibodies significantly decreased in six months after the acute phase [5]. Moreover, specific antibodies in mild patients were undoubtedly found to disappear more rapidly [6]. In addition, empirical findings from some studies showed that SARS-CoV-2-specific antibodies could offer protection against reinfection by providing the rationale for the administration of plasma containing SARS-CoV-2 neutralizing antibodies as a treatment for COVID-19[7, 8]. However, some case studies have also reported that people who recovered from COVID-19 can still be re-infected with SARS-CoV-2 in a relatively short time [9, 10]. This raised global concerns regarding how long the specific antibodies can last and function effectively within the body post-SARS-CoV-2 infection[11].

For people living with HIV(PLWH) infected with SARS-CoV-2, the clinical conditions may be more complicated for their immunodeficiency and immune dysregulation[12]. Published studies from Spain and our former study in Wuhan both showed that COVID-19 in PLWH might be more severe [13, 14]. But some current study findings tentatively suggest no difference in the incidence rate and adverse outcomes of COVID-19 between PLWH and the other individuals [15, 16]. A recent study proposed people with HIV in the UK seem to be at increased risk of COVID-19 mortality[17], but other researchers were skeptical about this statement[18]. In addition, there is very limited information on whether the immune response to SARS-CoV-2 infection is similar in PLWH and HIV-naïve individuals.

To fill this gap, we conducted a cohort study among both HIV infected and HIV-naïve COVID-19 patients in Wuhan, China, to understand the immune response among these individuals.

## Methods

### Study design and participants’ recruitment

Study participants consisted of COVID-19 patients who were admitted to the Zhongnan Hospital of Wuhan University and Wuhan NO.7 Hospital between January 15 and April 1, 2020. Patients were categorized into groups with HIV and without HIV. The diagnosis and classification of disease severity were defined based on the “New Coronavirus Pneumonia Prevention and Control Program (8th edition)” published by the National Health Commission of China [19].

### Laboratory procedures

Nucleic acid tests (NAT) for SARS-CoV-2 were conducted using real-time reverse transcriptional polymerase chain reaction (RT-PCR) kits as recommended by the Chinese center for disease control and prevention (CDC) following the WHO guidelines. Gold immunochromatography assay (qualitative test) was used in testing the IgG and IgM antibodies response against SARS-CoV-2 spike protein and nucleocapsid protein. All test kits used were approved by the China Food and Drug Administration and provided by Zhuhai Livzon Diagnostics Inc.

### Data collection and follow-up

Participants’ data were collected from January 15, 2020, including gender, age, comorbidities, smoking, lymphocyte count when illness onset, COVID-19 severity, and the time of disease diagnosis. PLWH participants’ data were acquired from the China CDCs’ AIDS Comprehensive Prevention and Control Data Information Management System. Required data and key information for HIV-naïve patients were acquired from their electronic clinical records. Comorbidities included hypertension, diabetes, coronary heart disease, tumors, and so on. All COVID-19 diagnosed patients were followed up regularly (every fifteen days) until November 30, 2020. NAT and antibody tests were done at each follow-up.

### Statistical analysis

Categorical variables were presented as counts (%), and continuous variables were presented as mean ± standard deviation (SD). Univariate and multivariable logistic regressions were used to identify factors associated with antibodies-positive rates. Gender, age, comorbidities, smoking, lymphocyte counts when illness onset, COVID-19 severity, and HIV status were included in the multivariable logistic regression model. Odds ratios (OR) with 95% confidence intervals (CI) and P-values were reported.

The Kaplan–Meier method was used to estimate the cumulative probability of IgG and IgM negative conversion and the median duration time of IgG and IgM. The Cox proportional hazards regression model was used to examine the factors associated with the duration time of IgG and IgM after controlling for confounders including gender, age, comorbidities, smoking, lymphocyte count when illness onset, COVID-19 severity, and HIV status. The adjusted hazard ratios (AHR) and 95% CI were calculated in the model. Statistical significance was defined as a two-sided p-value of less than 0.05. All statistical analyses were conducted using SPSS26.0.

## Results

### Characteristics of enrolled patients

In total, 203 COVID-19 patients were enrolled in the study, including 18 PLWH and 185 HIV-naïve individuals. The proportion of females in the HIV-naïve population was significantly higher than the PLWH group (62% VS 6%, P< 0.001), and the PLWHs group had a higher proportion of asymptomatic infected patients compared to the HIV-naïve group (33% VS 6%, P < 0.001). The positive conversion rates of IgG were 56% and 88% in the PLWH and the HIV-naïve group, respectively (P = 0.001) (Table 1).

### Antibody positive conversion rates and associated factors

**Figure 1:**
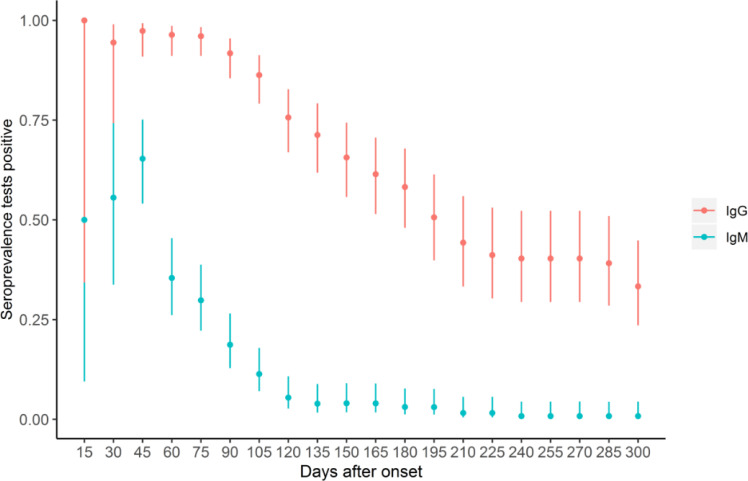
The variation trend of antibody positive rates among COVID-19 cases in Wuhan, China, 2020.

The positive conversion rate of IgG among asymptomatic carriers is 44%, which is significantly lower than the observed rate of 88% among moderate patients (adjusted OR (AOR) = 0.18, 95% CI: 0.05–0.65). We also found that although there was no statistically significant difference in IgM seroconversion between the two groups, PLWH group members had a lower IgG seroconversion rate compared to the HIV-naive group (AOR = 0.22, 95% CI: 0.05–0.90). The conversion rate of IgG was decreased with the increase of lymphocyte counts (AOR = 0.29, 95% CI: 0.09–0.90). There was no significant difference by age, gender, smoking conditions, and comorbidities in antibody-positive conversion groups (Table 2). The antibody-positive conversion rate reaches the peak at the 45th day after onset, then begins to decline (Fig. 1).

### Cumulative duration of antibodies and associated factors

The median duration time of IgG and IgM negative conversion since disease onset was 223 days (95% CI: 184-NA) and 112 days (95% CI: 107–116) respectively. The results of the Cox-proportional hazard regression model adjusted for confounders were shown in Table 3. With the increase of age, the duration time of IgM was increased(AHR = 0.96, 95% CI: 0.93–0.98). PLWH had a shorter IgG duration (AHR = 0.25, 95% CI: 0.09–0.70) and patients with higher lymphocyte counts at onset had a shorter IgG duration (AHR = 4.01, 95% CI 1.78–9.02).

## Discussion

Understanding the immune response towards SARS-CoV-2 among both PLWH and uninfected individuals is essential to providing tailored prevention and treatment measures against COVID-19. Findings from this study extend current literature by evaluating the similarities and differences in the immune response to SARS-CoV-2 infection between PLWH and HIV-naïve patients [3, 4, 6]. Compared to the HIV-naïve group, we found a lower positive conversion rate of IgG in PLWH, and the antibodies were lost much quicker.

Our findings showed that IgG positive conversion rate among COVID-19 infected individuals is higher in HIV-naïve patients (88%) than PLWH (56%). Similar to our findings, a study conducted in Iceland obtained 91% positive pan-immunoglobulin antibodies [2] and another in Chongqing of China reported an 84% IgG positive rate among HIV-naïve COVID-19 patients [20]. This observation is however not surprising as PLWH are likely to have imbalanced immune systems which could cause fewer antibody productions in the case of COVID-19 [21]. In addition, findings from other studies suggested that an impaired immune reactivity could contribute to low IgG positive[22]. Although this may suggest that PLWH may be more vulnerable with a SARS-CoV-2 infection, findings from other studies have observed no increased risk and severity of COVID-19 in affected PLWH [23]. Regardless, the risk of increased PLWH vulnerability should not be ignored as the reported similarity in immune response may only apply to PLWH. Therefore, the government in addition to implementing stronger prevention measures should improve antiretroviral therapy (ART) access for PLWH patients especially in the era of COVID-19. In addition, innovative means that promote retention in care and treatment adherence among PLWH are needed to achieve viral suppression are required.

We further discovered that the duration time for IgG positive conversion is shorter in PLWH. According to our findings, the positive conversion rates of IgG were 56% in PLWH and 88% in HIV-naïve patients respectively, and the peak was on the 45th day after COVID-19 onset. However, the positive rate of IgG dropped to 12% in PLWH and 33% among HIV-naïve individuals by the last observation time. In addition, a Cox proportional hazards regression conducted showed the IgG duration time in PLWH to be shorter than the general population. In explanation, findings from some case reports had shown specific antibodies response to be delayed or even vanish in PLWH with compromised immune status due to CD4 + T lymphocytes depletion and B lymphocytes dysfunction [24]. We, therefore, speculate that immune deficiency in PLWH could account for the low antibody response to SARS-CoV-2 infection and short cumulative duration time. The quick loss of antibodies may also imply a higher susceptibility to reinfections as previous studies have demonstrated the presence of IgG antibodies to be essential in reducing the risks of reinfection in the ensuing 6 months post treatment[25]. Some studies have however stipulated that ART may provide some form of protection against COVID-19 incidence given the effect some antiretroviral drugs have on the SARS-CoV-2 life cycle[26]. So, to positively enhance active immunity, encouraging treatment adherence is both important and urgent.

We found that the severity of COVID-19 was associated with the positive conversation rate of IgG, and lymphocyte counts at illness onset had an effect on both positive conversation rate and duration time of IgG. Our findings invariably showed a lower positive conversion rate of IgG in asymptomatic carriers from each patient group. This finding concurs with findings from previous studies conducted in Korea and Wuhan, China [27, 28]. Some studies have attributed that low level of viral loads that lead to low antibody response in asymptomatic individuals may have accounted for these findings [29, 30]. The attribution is plausibility as high viral loads such as those found in severe patients trigger a stronger antibody response. Through increased levels of viral antigens, there is a more significant depletion of lymphocyte [4] and a high positive conversion rate of antibodies in severe disease [31, 32], thus, result in a longer IgG duration in patients with lower lymphocyte counts at onset.

Our study includes some limitations. First, because of the fundamental lack of an antibody detection kit in the early days of the SARS-CoV-2 epidemic in Wuhan, the possible opportunity for early antibody testing lacked. This however exerts no direct influence on our comparison of immune response between the two patient groups. Second, the specific number of Covid-19 infected PLWH was relatively small in our study. For this reason, our study was naturally limited in its power to detect differences among COVID-19 patients with different HIV statuses. Third, due to the limited information collected in this study, many of the potential confounders were not adjusted which may bias our results.

## Conclusion

In conclusion, our study revealed that the positive conversion rate of the SARS-Cov-2 specific antibodies was relatively lower and quickly lost in PLWH with COVID-19. This meant PLWH was in a disadvantaged situation when affected with COVID-19 disease, and they may be more susceptible to reinfections.

## Figures and Tables

**Table 1:** Characteristics of enrolled patients with COVID-19, Wuhan, China, 2020 (N = 203)

	HIV negative(n = 185)		HIV positive(n = 18)		P value
	n	%	n	%	
**Age, years**	45.83 ± 1.02		44.93 ± 2.46		0.75
**Gender**					
Male	71	38.38	17	94.44	< .001
Female	114	61.62	1	5.56	
**Comorbidities**					
No	156	84.32	12	66.67	0.09
Yes	29	15.68	6	33.33	
**Smoking**					
No	149	80.54	17	94.44	0.21
Yes	36	19.46	1	5.56	
**Lymphocyte count (10^9^/L)**	1.21 ± 0.04		1.08 ± 0.10		0.72
**Types of COVID-19**					
Asymptomatic carriers	12	6.48	6	33.33	< 0.001
Mild	4	2.16	0	0.00	
Moderate	165	89.19	9	50.00	
Severe	16	8.65	3	16.67	
**Positive conversion of IgM**	96	51.89	6	33.33	0.15
**Positive conversion of IgG**	163	88.11	10	55.56	0.001

Data are presented as count (%) or mean ± standard deviation.

**Table 2:** The antibody positive conversion rates and associated factors among COVID-19 cases in Wuhan, China, 2020 (N = 203)

Variables	IgM (AOR and 95% CI) *	IgG (AOR and 95% CI) *
**Age**	0.99(0.97–1.02)	1.03(0.98–1.07)
**Gender**		
Male	*Ref*	*Ref*
Female	1.20(0.56–2.56)	1.13(0.35–3.66)
**Current smoking**	0.56(0.24–14.14)	1.36(0.12–15.27)
**Comorbidities**	1.12(0.44–2.85)	0.39(0.10–1.55)
**Type**		
Moderate	*Ref*	*Ref*
Asymptomatic	0.34(0.08–1.35)	0.18 (0.05–0.65)
Mild	0.68(0.06–8.27)	2.71×10^8^(0.00-NA)
Severe	2.43(0.70–8.45)	0.42(0.08–2.16)
**Co-infected with HIV**	0.64(0.19–2.19)	0.22(0.05–0.90)
**Lymphocyte counts(10^9^/L)**	0.48(0.22–1.08)	0.29(0.09–0.90)

Note:

* Each association was mutually adjusted for the other characteristics in the table.

**Table 3:** The associated factors of IgG and IgM duration time among COVID-19 cases in Wuhan, China, 2020 (N = 203)

Variables	IgM (AHR and 95% CI) *	IgG (AHR and 95% CI) *
**Age**	0.96(0.93–0.98)	1.25(0.53–2.91)
**Gender**		
Male	*Ref*	*Ref*
Female	1.47(0.71–3.04)	0.99(0.96–1.00)
**Current smoking**		
No	*Ref*	*Ref*
Yes	0.69(0.19–2.58)	0.83(0.09–7.24)
**Comorbidities**		
No	*Ref*	*Ref*
Yes	1.23(0.55–2.72)	1.29(0.33–5.05)
**COVID-19 severity**		
Moderate	*Ref*	*Ref*
Mild	0.79(0.09–6.84)	0.65(0.14–2.94)
Severe	1.57(0.67–3.68)	0.85 (0.18–3.98)
**Co-infected with HIV**		
No	*Ref*	*Ref*
Yes	1.23(0.44–3.44)	0.25(0.09–0.70)
**Lymphocyte(10^9^/L)**	0.86(0.33–2.24)	4.01(1.78–9.02)

Note:

* Each association was mutually adjusted for the other characteristics in the table.
